# Supplementary material for: A Pilot Study on the Effects of Transcranial Direct Current Stimulation on Brain Rhythms and Entropy during Self-Paced Finger Movement using the Epoc Helmet
Source: Front Hum Neurosci. 2017 Apr 25;11:201. doi: 10.3389/fnhum.2017.00201 (PMC5408787; doi:10.3389/fnhum.2017.00201)
Supplement: Supplementary file 1 [file Data_Sheet_1.zip › Complementary_results/Power-Spectral-Entropy_average_PSD_windows/PSE_mean-win_F4.pdf]

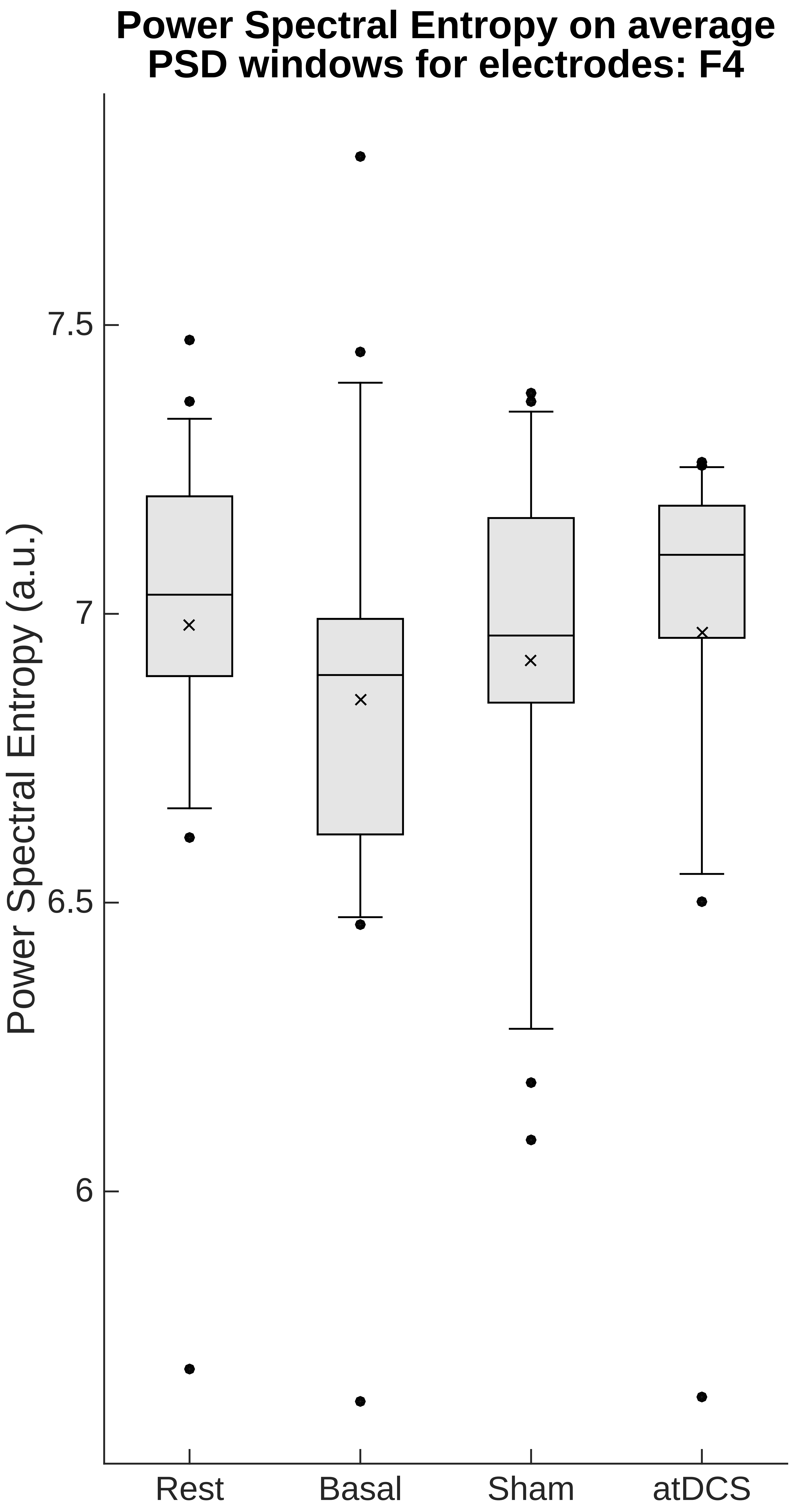
Power Spectral Entropy on average
PSD windows for electrodes: F4
Power Spectral Entropy (a.u.)
7.5
7
6.5
6
Rest
Basal
Sham
atDCS
